# Upregulation of Parkin Accelerates Osteoblastic Differentiation of Bone Marrow-Derived Mesenchymal Stem Cells and Bone Regeneration by Enhancing Autophagy and β-Catenin Signaling

**DOI:** 10.3389/fcell.2020.576104

**Published:** 2020-09-15

**Authors:** Wei Zhang, Weiduo Hou, Mo Chen, Erman Chen, Deting Xue, Chenyi Ye, Weixu Li, Zhijun Pan

**Affiliations:** ^1^Department of Orthopedics, The Second Affiliated Hospital, School of Medicine, Zhejiang University, Hangzhou, China; ^2^Orthopaedics Research Institute, Zhejiang University, Hangzhou, China; ^3^Department of Rheumatology, The Second Affiliated Hospital of Zhejiang University, School of Medicine, Hangzhou, China

**Keywords:** Parkin, osteogenic differentiation, stem cells, autophagy, beta-Catenin

## Abstract

Osteogenic differentiation of bone marrow-derived mesenchymal stem cells (BMSCs) plays a key role in bone formation. Parkin, an E3 ubiquitin ligase, related to Parkinson’s disease and aging. Previous studies have indicated that Parkinson’s disease have a higher risk of osteoporotic fracture. To investigate the effects and underlying mechanism of Parkin in the osteogenic differentiation of BMSCs, osteogenic differentiation was analyzed following upregulation or downregulation of Parkin. We found that Parkin was increased during differentiation. Parkin overexpression enhanced osteo-specific markers, and downregulation of Parkin mitigated osteo-specific markers. Moreover, upregulation of Parkin promoted β-catenin expression and autophagy and vice versa. The upregulation of β-catenin enhanced autophagy, and the activation of autophagy also increased the expression of β-catenin in *Parkin*-downregulated BMSCs. Parkin-overexpressed cell sheets accelerated bone healing in a tibial fracture model. Based on these results, we concluded that Parkin meditates osteoblastic differentiation of BMSCs via β-catenin and autophagy signaling.

## Introduction

In clinical settings, fractures fail to heal in 5–10% of patients (Zura et al., 2016). Bone marrow-derived mesenchymal stem cells (BMSCs) have the potential to differentiate into bone tissue, making them attractive candidates for bone regeneration (Walmsley et al., 2016). Therefore, it is important to identify therapeutic strategies to enhance BMSC osteogenesis.

Parkin, also named Park2, is an E3 ubiquitin ligase (Park et al., 2017). Parkin mutations result in autosomal recessive Parkinson’s disease (Lücking et al., 1998; Pickrell and Youle, 2015). Parkin is widely expressed in various cells and tissues and plays a critical role in many biological processes, including cell growth, apoptosis, differentiation, inflammation, and autophagy (Olzmann et al., 2007; Callegari et al., 2017; Jung et al., 2017; Wang et al., 2018). As a protective protein, Parkin activates mitochondrial quality control pathways and enhances autophagy (Seirafi et al., 2015). Parkin also regulates chondrocyte survival (Ansari et al., 2018) and is required to regulate exercise signaling in muscles during aging (Chen et al., 2018). Interestingly, patients with Parkinson’s disease have a higher risk of osteoporotic fracture (Malochet-Guinamand et al., 2015). BMSCs from patients with Parkinson’s disease showed impaired differentiation into adipocytes (Angelova et al., 2018), suggesting the expression of Parkin may be linked to the differentiation of BMSCs. However, the roles of *Parkin* during osteogenesis have not yet been elucidated.

In this study, we investigated the effects of Parkin on osteogenic differentiation of BMSCs. We found that upregulation of Parkin enhanced the osteogenic differentiation of BMSCs both *in vitro* and *in vivo*.

## Materials and Methods

### Reagents and Cells

This study was carried out in accordance with the principles of the Basel Declaration and the recommendations of Zhejiang University. The protocol was approved by the Animal Ethics Committee of Zhejiang University. Mouse BMSCs from Balb/c were purchased from Cyagen Biosciences Inc. (MUCMX-9001, Guangzhou, China).

### Adenoviral Vectors

Parkin overexpression adenovirus vectors (OE; AV-CMV > Mouse Parkin/T2A/PolyA) and negative control adenovirus vectors (NC; AV-CMV > PolyA) were purchased from Cyagen Biosciences Inc., and the viral titers were l × 10^10^ PFU/mL. mRNA and protein levels of Parkin were analyzed by quantitative real-time polymerase chain reaction (RT-qPCR) and western blot (WB) analysis, respectively.

### Small Interfering RNA (siRNA)

Three siRNAs targeted to *Parkin* and a negative control siRNA were designed and purchased from GenePharma Inc. (Shanghai, China). The sequences are listed in Table 1. Lipo6000^TM^ transfection reagent (C0526, Beyotime Biotechnology, Shanghai, China) in Opti-MEM^TM^ I Reduced Serum Medium (51985091, Thermo Fisher Technology Co., Ltd., China) was used for transfection according to the manufacturer’s instructions. The levels of Parkin were analyzed by WB.

**TABLE 1:** Small interfering RNA sequences to Parkin and negative control.

	5′-3′
shRNA1	GGAUCAGCAGAGCAUUGUUTT AACAAUGCUCUGCUGAUCCTT
shRNA2	GCUCCAUCACUUCAGGAUUTT AAUCCUGAAGUGAUGGAGCTT
shRNA3	CCUUCUGCCGGGAAUGUAATT UUACAUUCCCGGCAGAAGGTT
shRNA negative control	UUCUCCGAACGUGUCACGUTT ACGUGACACGUUCGGAGAATT

### Cell Counting Kit-8 (CCK-8)

To analyze the cytotoxic effects of Parkin on the viability of BMSCs, the cells were treated with 10% CCK-8 (Dojindo, Kumamoto, Japan) in low-glucose Dulbecco’s Modified Eagle’s Medium without fetal bovine serum for 2 h in 5% CO_2_ at 37°C. Absorbance at 450 nm was measured using a microplate reader (ELX808; BioTek, Winooski, VT, United States).

### Osteoblastic Differentiation of BMSCs

BMSCs were cultured in complete growth medium (MUBMX-90011, Cyagen Biosciences) and incubated at 37°C under 5% CO_2_. For osteogenic differentiation, the cells were cultured in osteogenic induction medium (MUBMD-90021; Cyagen Biosciences). The cells were maintained by the addition of fresh osteogenic induction medium every 2–3 days.

### Alizarin Red Staining (ARS)

Following induction of osteogenic differentiation, mineral deposition was assessed by ARS (Cyagen Biosciences). Cells were fixed in 4% paraformaldehyde (Sangon Biotech, Shanghai, China) for 15 min at room temperature and washed with distilled water twice. A 1% solution of Alizarin Red was added and incubated for 10 min at room temperature, followed by rinsing with distilled water. Images were then taken using an inverted microscope (Leica, Wetzlar, Germany).

### Oil Red O Staining

According our previous study (Hou et al., 2019), the fat droplet was evanulated by Oil Red O staining. Briefly, after the induction of adipogenesis, cells were fixed and then washed with PBS. An Oil Red O staining solution was incubated for 15–30 min. For quantitation, Oil Red O was dissolved by isopropanol and then the absorbance at 500 nm was measured.

### Immunofluorescence (IF) Analysis

Cells were cultured in a 12-well plate. Microtubule-associated protein 1 light chain 3 (LC3; 14600-1-AP, ProteinTech, Wuhan, Hubei, China) and activated β-catenin were detected using a fluorescence microscope (EU5888; Leica). Briefly, after treatment, cells were fixed in 4% paraformaldehyde for 15 min. Next, they were blocked in 5% bovine serum albumin for 30 min and incubated overnight with anti-LC3 (1:400; Cell Signaling Technology, Shanghai, China) and activated β-catenin (1:100; Cell Signaling Technology) at 4°C. The next day, cells were washed with phosphate-buffered saline and incubated with a fluorescence-conjugated secondary antibody (Abcam, Shanghai, China) for 2–3 h at 37°C, and nuclei were stained with 4’,6-diamidino-2-phenylindole (DAPI; Sigma-Aldrich, Shanghai, China) for 2 min. Cells were observed using an inverted fluorescence microscope (Leica).

### RT-qPCR

Total cellular RNA was isolated and reverse-transcribed into cDNA according to our previous study (Zhang W. et al., 2018) and 1 μL of cDNA was used as the template for the qPCR reaction. All gene transcripts were quantified by PCR using the Power SYBR^®^ Green PCR Master Mix (Takara Bio, Kusatsu, Japan) on the ABI StepOnePlus System (Applied Biosystems, Warrington, United Kingdom). mRNAs of the target genes and the housekeeping gene (glyceraldehyde 3-phosphate dehydrogenase [*GAPDH*]) were quantified in separate tubes. All primers were synthesized by Sangon Biotech. The primers used were as follows: *Gapdh*, Forward: CGACTTCAACAGCAACTCCCACTCTTCC; Reverse: TGGGTGGTCCAGGGTTTCTTACTCCTT; Alp, Forward: CCAACTCTTTTGTGCCAGAGA; Reverse: CGCC TGGTAGTTGTTGTGAGCATAG; *Runx2*, Forward: GACTG TGGTTACCGTCATGGC; Reverse: GGCTACATTGGTGTT GAGCTTTT; *Col1*, Forward: CCACGTCTCACCATTGGGG; Reverse: GCTCCTCTTAGGGGCCACT; and *Parkin* Forward: TCTTCCAGTGTAACCACCGTC; Reverse: GGCAGGGAGTAGCCAAGTT. The PCR conditions were as follows: 95°C for 30 s, 40 cycles of 95°C for 5 s and 60°C for 30 s. The relative target gene expression levels were calculated using the 2^–^^Δ^ΔCt method.

### Western Blot Analysis

Cells were lysed in RIPA lysis buffer (Beyotime Biotechnology, Shanghai, China) with a protease and phosphatase inhibitor cocktail kit (P1048; Beyotime Biotechnology). Nuclear and cytoplasmic proteins were separated by a nuclear and cytoplasmic protein extraction kit according to the manufacturer’s instructions and a previous study (Zhang et al., 2012) (P0027; Beyotime Biotechnology).

Proteins were separated via 8–12% sodium dodecyl sulfate-polyacrylamide gel electrophoresis and transferred to a polyvinylidene fluoride membrane (Millipore, Shanghai, China). After blocking in 5% non-fat milk for 2 h, the membranes were incubated overnight at 4°C with antibodies specific for GAPDH (1:5000; 10494-1-AP; ProteinTech Inc., Wuhan, China), RUNX2 (1:1000; #12556; Cell Signaling Technology), Parkin (1:500; 14060-1-AP; ProteinTech Inc.); collagen I (COL1A1, 1:1000; ab34710, Abcam), sequestosome 1 (p62/SQSTM1, 1:2000; 18420-1-AP; ProteinTech Inc.), LC3 (1:1000; #4108; Cell Signaling Technology), Axis inhibition protein 2 (AXIN2; 1:1000; #2151, Cell Signaling Technology), LEF1(1:1000; #2230, Cell Signaling Technology) or non-phospho (active) β-catenin (1:1000; #19807; Cell Signaling Technology). Horseradish peroxidase-conjugated goat anti-rabbit IgG (1:5000; Boster Biological Technology, Pleasanton, CA, United States) was used as a secondary antibody for 2 h at room temperature. The immunoreactive bands were detected using an enhanced chemiluminescent detection reagent (Millipore, Shanghai, China). Signal intensity was measured using a Bio-Rad XRS chemiluminescence detection system (Bio-Rad, Hercules, CA, United States).

### *In vivo* Evaluation of Rats

The bone-forming ability of Parkin was assessed in a tibial fracture model in Sprague–Dawley rats in accordance with our previous studies (Zhang et al., 2016; Ye et al., 2018; Zhang W. et al., 2018). All experiments were conducted in accordance with the Animal Care and Use Committee guidelines of Zhejiang province and the Institutional Animal Care and Use Committee of Zhejiang University. Three-month-old male (approximately 200 g) Sprague–Dawley rats were obtained from the Academy of Medical Sciences of Zhejiang province. The cell sheets were fabricated as previously described (Zhang et al., 2016; Zhang W. et al., 2018). The tibial fracture model was established as our previously reports (Zhang et al., 2016; Zhang W. et al., 2018). Rats were induced by inhalation of 2–5% isoflurane and maintained with 2% isoflurane inhalation during the surgery (Zhang et al., 2016; Zhang W. et al., 2018). An intramedullary needle (1.2-mm diameter stainless steel syringe needle) was inserted inside the medullary canal of the right tibia. An osteotomy with a transverse 1 mm wide were performed by an electronic saw. The 15 tibial fractures in 15 rats were randomized into three groups. In the blank group (*n* = 5), nothing was grafted into the fracture site; in the NC group (*n* = 5), a sheet of lenti-control BMSCs was wrapped around the fracture site; and in the OE group (*n* = 5), a sheet of BMSCs overexpressing Parkin was grafted around the fracture site. After surgery, the rats received subcutaneous injections of 0.05 mg/kg buprenorphine for analgesia.

The rats were sacrificed via CO_2_ administration at 8 weeks after surgery according to the American veterinary medical association euthanasia guidelines.

### Microcomputed Tomography (Micro-CT) Evaluation

To evaluate callus formation and bridging bone formation at fracture sites 8 weeks postoperatively, the samples were scanned using the μCT-100 imaging system (Scanco Medical, Brüttisellen, Switzerland) with X-ray energy settings of 70 kVp, 1024 reconstruction matrix, 14.8 μm slice thickness, and an exposure time of 300 ms. The bone volume fraction was calculated by three-dimensional standard microstructural analysis. Bone volume (BV), total volume (TV), and bone volume fraction (BV/TV) were measured in the region of the fracture as in previous studies (Zhang et al., 2016; Chen et al., 2017; Zhang W. et al., 2018).

### Histological Evaluation

Samples were fixed with 4% paraformaldehyde for 48 h at 4°C and decalcified using 10% EDTA (Sigma-Aldrich) with a solution change once weekly for at least 8 weeks at 4°C before embedding in paraffin. Serial sections with a thickness of 3 μm were cut and mounted onto adhesion microscope slides (Ref. 188105, Citoglas, Beijing, China). Hematoxylin-eosin and Masson’s trichrome staining were carried out separately on consecutive tissue sections, as described in our previous study (Zhang et al., 2016). According to pervious study (Sawa et al., 2018), the numbers of osteoblast were counted in the middle of the fracture callus under the microscope 100 times the field of view. Bone, fibrous and cartilage tissues were semi-quantified in the fracture healing part by ImageJ software (Minear et al., 2010; Hu and Olsen, 2016).

### Statistical Analysis

Statistical analysis was performed using SPSS statistical software for Windows, version 19.0 (IBM, Armonk, NY, United States). All experiments were performed in at least triplicate, and the data are presented as the mean ± standard deviation. Statistical significance was determined using a two-tailed Student’s *t*-test when comparing two groups. A value of *P* ≤ 0.05 was considered statistically significant.

## Results

### Levels of Parkin Increased During Osteoblastic Differentiation of BMSCs

**FIGURE 1:**
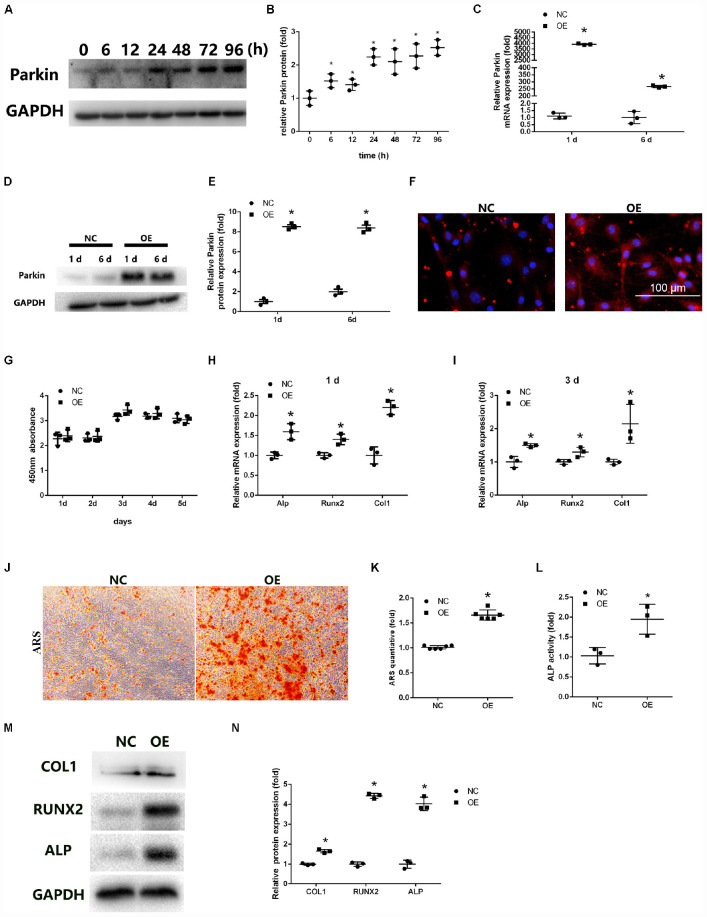
Overexpression of Parkin enhances osteoblastic differentiation of bone marrow mesenchymal stem cells (BMSCs). **(A,B)** Protein expression levels of Parkin were determined at 0, 6, 12, 24, 48, 72, and 96 h after induction of osteoblastic differentiation. **(C)** mRNA expression of Parkin was analyzed by quantitative real-time polymerase chain reaction (RT-qPCR) at days 1 and 6 after infection. **(D,E)** Protein expression of Parkin was analyzed by western blot (WB) analysis at days 1 and 6 after infection. **(F)** Protein expression of Parkin was analyzed by immunofluorescence staining at day 6 after infection. **(G)** Proliferation was detected by Cell Counting Kit-8 in the Parkin-overexpressed and control group at days 1, 2, 3, 4, and 5. **(H)** Osteo-specific genes Alp, Runx2, and Col1 were analyzed by RT-qPCR at day 1 after osteogenic induction. **(I)** Osteo-specific genes Alp, Runx2, and Col1 were analyzed by RT-qPCR at day 3 after osteogenic induction. **(J,K)** Mineral deposits were determined by Alizarin Red staining at day 12 after osteogenic induction. **(L)** Alkaline phosphatase activity at day 3 of osteogenic differentiation of BMSCs. **(M,N)** Osteo-specific proteins Alp, Runx2, and Col1 were analyzed by WB at day 3 after osteogenic induction. Data are expressed as the mean ± standard deviation (SD) (*n* = 3). NC, BMSCs transfected with negative control adenovirus vectors; OE, BMSCs transfected with Parkin-overexpressed adenovirus vectors; *P < 0.05 vs. BMSCs in the NC group.

To investigate the levels of Parkin, WB analysis was performed at 0, 6, 12, 24, 48, 72, and 96 h after the induction of differentiation. Protein expression of Parkin gradually increased during differentiation (Figures 1A,B).

### Parkin Overexpression Enhanced Osteoblastogenesis of BMSCs

We used adenoviral vectors to establish Parkin-overexpressing BMSCs. RT-qPCR and WB analysis revealed that the expression of Parkin was upregulated in BMSCs at days 1 and 6 after lentiviral vector infection (Figures 1C–E). IF analysis also showed that the levels of Parkin markedly increased with the overexpression vectors (Figure 1F).

The cytotoxic effects of Parkin overexpression on BMSCs were evaluated using the CCK-8 assay. No significant adverse effects were observed between the OE and NC groups (Figure 1G).

Osteo-specific markers, including Alp, Runx2, and Col1, were detected by RT-qPCR and WB analysis following induction of osteogenic differentiation. The results indicated the mRNA levels of osteo-specific markers were significantly increased at days 1 and 3 (Figures 1H,I). Protein levels of ALP, RUNX2, and COL1 were also upregulated in BMSCs (Figures 1M,N).

ALP activity was significantly higher in Parkin-overexpressed cells than in the control group at day 3 after osteogenic induction (Figure 1L). Mineral deposits were also assessed at day 12 after induction of osteogenic differentiation. ARS showed that mineral deposits were significantly increased in Parkin-overexpressed cells (Figures 1J,K). Moreover, oil Red O staining showed significantly less fat droplet in the OE group (Supplementary Material S1).

### Downregulation of *Parkin* by siRNA Compromised Osteoblastogenesis of BMSCs

**FIGURE 2:**
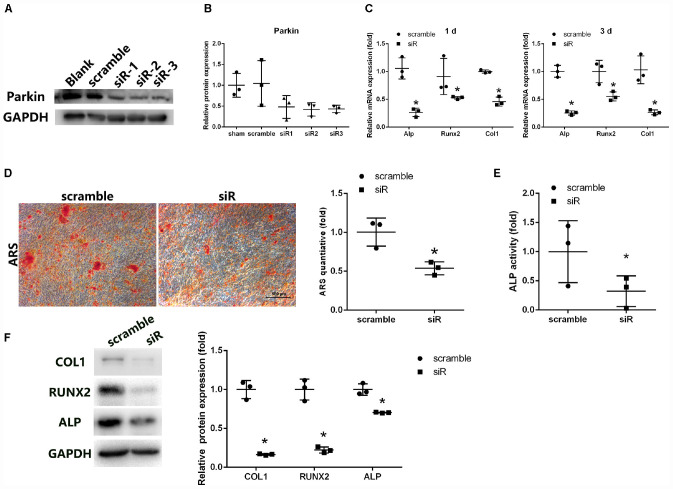
Parkin knockdown suppresses osteogenic differentiation of BMSCs. **(A,B)** Protein levels of Parkin were analyzed by WB analysis at day 3 after small-interfering RNA treatment. **(C)** Osteo-specific genes Alp, Runx2, and Col1 were analyzed by RT-qPCR at days 1 and 3 after osteogenic induction. **(D)** Mineral deposits were determined by Alizarin Red staining at day 12 after osteogenic induction. **(E)** Alkaline phosphatase activity was determined at day 3 of osteogenic differentiation of BMSCs. **(F)** Expression levels of COL1, RUNX2, and ALP were determined by WB analysis at day 3 of osteogenic differentiation. Protein expression levels were normalized to glyceraldehyde-3-phosphate dehydrogenase (GAPDH). Data are expressed as the mean ± SD of three independent experiments, and one of three independent experiments is shown. *P < 0.05 vs. BMSCs treated with negative control siRNA.

At day 2 after siRNA transfection, the expression of Parkin was examined by WB. Expression of Parkin was downregulated by siRNA, especially siRNA3 (Figures 2A,B). Thus, siRNA3 was selected for subsequent experiments.

Expression of osteo-specific markers (Alp, Runx2, and Col1) was detected by PCR and WB analysis following induction of osteogenic differentiation. mRNA levels of Alp, Runx2, and Col1 were significantly reduced following treatment with Parkin-siRNA (Figure 2C). Protein levels of ALP, RUNX2, and COL1 were also downregulated in the Parkin siRNA group (Figure 2F). Moreover, fewer mineral deposits were observed following downregulation of Parkin (Figure 2D). Even before osteoblastic induction at the start of the culture (0 h), the expression levels of these osteo-specific genes were significantly decreased. More fat droplet were found in the Parkin-downregulating BMSCs (Supplementary Material S1).

### Overexpression of Parkin Upregulated β-Catenin Expression and Increased Autophagy and *Parkin* Deletion Decreased β-Catenin and Autophagy

**FIGURE 3:**
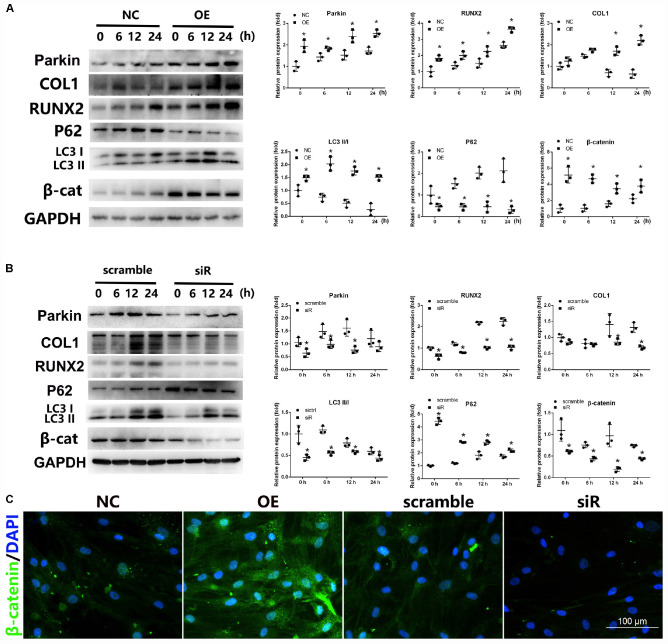
Expression of Wnt/β-catenin and autophagy signaling after upregulation or downregulation of Parkin during osteogenic differentiation of BMSCs. **(A)** When Parkin was overexpressed, protein levels of Parkin, COL1, RUNX2, LC3, p62, β-catenin, and GAPDH were determined by WB analysis after osteogenic differentiation for 0, 6, 12, and 24 h. Protein expression levels were normalized to GAPDH. Data are expressed as the mean ± SD of three independent experiments, and one of three independent experiments is shown. **(B)** When *Parkin* was downregulated by siRNA, the expression levels of Parkin, COL1, RUNX2, LC3, p62, β-catenin, and GAPDH protein were determined by WB analysis after osteogenic differentiation for 0, 6, 12, and 24 h. Protein expression levels were normalized to GAPDH. Data are expressed as the mean ± SD of three independent experiments, and one of three independent experiments is shown. **(C)** Expression of β-catenin was analyzed by immunofluorescence staining at day 1 after osteogenic induction. *P < 0.05 vs. BMSCs treated with osteogenic induction medium alone. Magnification × 40.

To determine the underlying mechanisms of Parkin during osteoblastic differentiation, Wnt/β-catenin signaling and autophagy were investigated. WB analysis revealed that overexpression of Parkin significantly upregulated the expression of β-catenin (Figure 3A). Markers of autophagy, LC3 II/I ratio was activated and p62 was inhibited by upregulation of Parkin (Figure 3A). Interestingly, Parkin knockdown significantly inhibited the levels of β-catenin and LEF1 (Figure 3B, Supplementary Material S2). AXIN, a negative regulator of wnt/β-catenin signaling pathway, were upregulated in the Parkin deletion group (Supplementary Material S2). Furthermore, LC3 II/I rate decreased and p62 increased after Parkin knockdown (Figure 3B).

IF analysis was also performed to investigate the influence of Parkin; the results revealed that Parkin overexpression increased the expression of β-catenin while Parkin knockdown significantly decreased β-catenin (Figure 3C).

### Activation of β-Catenin Upregulated Autophagy, and Activation of Autophagy Enhanced the Expression of β-Catenin in Parkin-Downregulated BMSCs

**FIGURE 4:**
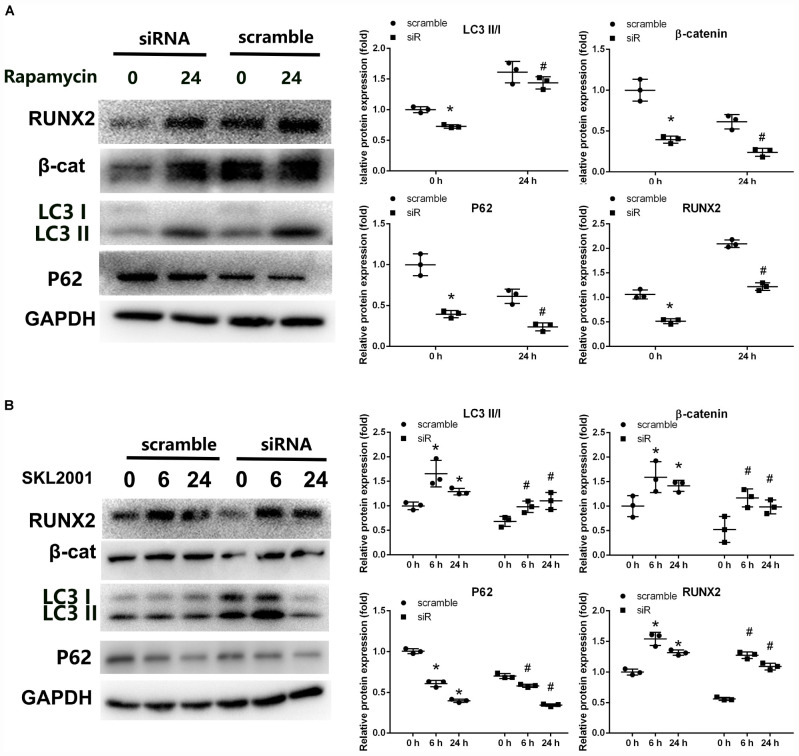
Crosstalk between β-catenin signaling and autophagy in Parkin-downregulated BMSCs. **(A)** Expression levels of RUNX2, β-catenin, LC3, p62, and GAPDH were determined by WB analysis following rapamycin treatment (20 nM, 24 h). Protein expression levels were normalized to GAPDH. Data are expressed as the mean ± SD of three independent experiments, and one of three independent experiments is shown. *P < 0.05 vs. group with scramble siRNA. ^#^*P* < 0.05 vs. group with Parkin small-interfering RNA. **(B)** Expression levels of RUNX2, β-catenin, LC3, p62, and GAPDH were determined by WB analysis following treatment with SKL2001 (20 μM, 6 and 24 h). Protein levels were normalized to GAPDH. Data are expressed as the mean ± SD of three independent experiments, and one of three independent experiments is shown. *P < 0.05 vs. group with scramble siRNA. ^#^*P* < 0.05 vs. group with Parkin siRNA.

To study the crosstalk between autophagy and Wnt/β-catenin signaling involved in the regulation of Parkin, specific agonists of the relevant pathway were used. To investigate whether the decreased autophagy in Parkin-downregulated BMSCs is responsible for the downregulation of β-catenin, we used rapamycin (20 nM, 24 h) to upregulate autophagy (Wan et al., 2017; Choi et al., 2018). In response to rapamycin, a significant upregulation of β-catenin expression was observed in addition to autophagy activation (Figure 4A). SKL2001 is a specific agonist of the Wnt/β-catenin signaling pathway. According to a previous study (Zhou et al., 2019), the expression of LC3 and p62 were analyzed by WB analysis after 6 and 24 h of stimulation with SKL2001 (20 μM). The LC3 II/I ratio was significantly increased with the upregulation of β-catenin following treatment of Parkin-downregulated BMSCs with SKL2001 (Figure 4B). p62 expression was also downregulated following β-catenin activation (Figure 4B). These results indicate there is crosstalk between Wnt/β-catenin and autophagy signaling.

### Parkin-Overexpressed Cell Sheets Accelerated Bone Healing in a Tibial Fracture Model in Rats

To examine the effect of Parkin on osteogenesis of BMSCs *in vivo*, overexpressed BMSC sheets were used (Zhang et al., 2016, 2018; Chen et al., 2017) and micro-CT and histologic analyses were performed.

**FIGURE 5:**
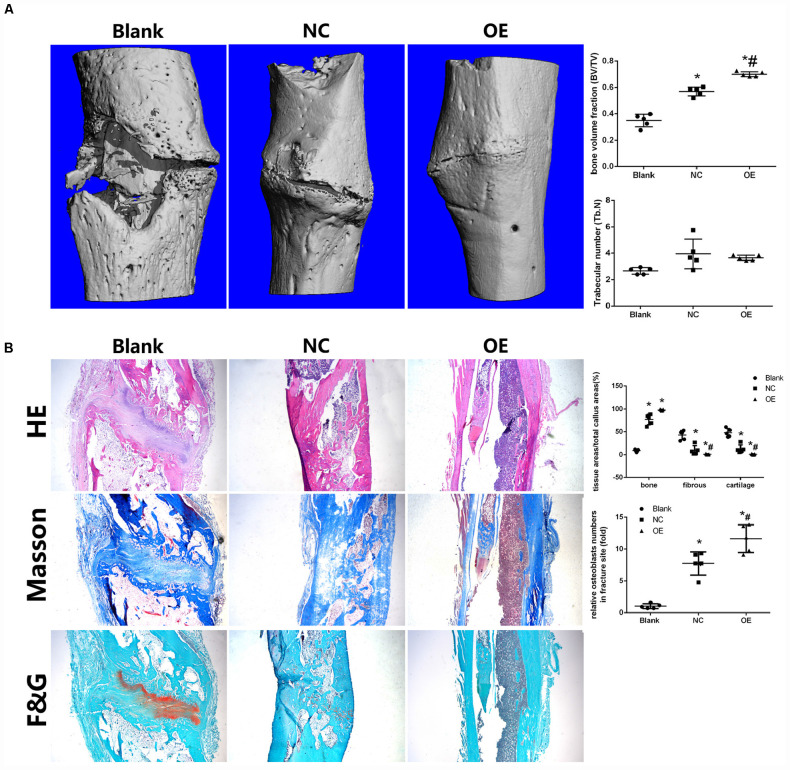
Parkin-overexpressed cell sheets accelerate bone formation in a tibial fracture model in rats. **(A)** Microcomputed tomography (micro-CT) analysis of bone healing. Bone volume and trabecular thickness were analyzed by micro-CT. Data are expressed as the mean ± SD. *P < 0.05 vs. fractures alone group (Blank). ^#^*P* < 0.05 vs. fractures with the negative overexpressing-cell sheet (NC). **(B)** Histologic analysis for bone healing. HE, hematoxylin and eosin staining; Masson, Masson’s trichrome staining, F&G, fast green and Safranin staining. Magnification × 40. Data are expressed as the mean ± SD. *P < 0.05 vs. fractures alone group (Blank). ^#^*P* < 0.05 vs. fractures with the negative overexpressing-cell sheet (NC).

Micro-CT demonstrated improved bone healing in the *Parkin*-overexpressing group compared with the control and blank groups (Figure 5A). A marked increase in bone volume fraction was observed in the OE group compared with the other two groups. There was no significant difference in trabecular thickness among the three groups (Figure 5A).

Histologic analysis demonstrated that the fractures in the blank group were filled with substantial fibroblasts and some chondrocytes in fracture sites. No continuous callus was observed. In the NC group, there was a thick newly woven bone tissue at the fractures area with few chondrocytes. In the OE group, the fractures were bridged with continuous cortical bone. The fracture sites were almost sealed, and the remodeling of the callus was complete (Figure 5B). Moreover, bone, fibrous tissues, and cartilage were quantified and the osteoblasts were counted in the fracture healing part. The results showed that the OE group had a significantly lower ratio in fibrous tissues and cartilage compared with the other two groups (Figure 5B). The number of osteoblasts in the fracture sites were higher in the OE group (Figure 5B). IF in the fracture callus tissues revealed significantly more β-catenin in OE group than in the NC group. A significant decrease in p62 was found with fracture sites treated with the OE sheet compared with the other two groups (Supplementary Material S3).

## Discussion

This is the first study to investigate the effects of Parkin on osteogenic differentiation of BMSCs. We found that Parkin expression gradually increased during osteogenic differentiation. Notably, before osteoblastic induction, the expression levels of specific osteogenic genes were significantly decreased by Parkin deletion. Moreover, Parkin overexpression enhanced the specific osteogenic genes before osteoblastic induction, indicating that BMSCs overexpressing Parkin had acquired an intrinsic osteoblast phenotype. Parkin overexpression accelerated osteoblastic differentiation and mitigates adipogenic phenotype. Moreover, Parkin deletion impaired osteoblastic differentiation and enhances adipogenesis. Osteogenesis and adipogenesis are always exclusive during BMSCs differentiation (Bian et al., 2016). Accumulating evidences have revealed that the unbalance between them results in the disorder of bone regeneration. These results indicate that Parkin involved in stem cells lineage commitment switching between osteogenesis and adipogenesis, which acts a positive regulator in the osteoblastic differentiation of BMSCs.

The Wnt signaling pathway plays a critical role in bone metabolism (Tu et al., 2015; Zhang et al., 2016; Chen et al., 2017; Zhang W. et al., 2018), which includes the canonical signaling pathway and non-canonical pathways based on the role played by β-catenin. Our previous studies also revealed that activation of the Wnt/β-catenin signaling pathway promoted osteoblastic differentiation of BMSCs (Zhang et al., 2016; Chen et al., 2017; Zhang W. et al., 2018). β-Catenin is an essential downstream molecule of the canonical Wnt cascade (Duan and Bonewald, 2016). In the Wnt inactive state, cytosolic β-catenin is phosphorylated and degraded (Sakanaka, 2002) whereas in the Wnt active state, the Wnt ligand prevents β-catenin phosphorylation and non-phosphorylated β-catenin transduces cytosolic signals to the nucleus and triggers transcription of target genes (Sakanaka, 2002; Valenta et al., 2012; Marchetti, 2018). Axin2 negatively regulates β-catenin and is a target of Wnt/β-catenin signaling (Bernkopf et al., 2019). LEF1, binding to Wnt response elements to provide docking sites for β-catenin is a Wnt signaling pathway marker (Zhang M. et al., 2018). A growing body of evidence suggests that susceptibility genes of Parkinson’s disease are associated with Wnt signaling regulation (Berwick and Harvey, 2014; L’Episcopo et al., 2014). Rawal et al. (2009) revealed that Parkin could prevent dopaminergic neuronal death through regulation of the Wnt signaling pathway. In this study, to investigate the relationship between Wnt signaling and Parkin, the levels of active β-catenin were analyzed. Upregulation of Parkin was shown to promote β-catenin activation and trigger the expression of osteo-specific genes. Moreover, depletion of Parkin led to decreases in β-catenin levels.

**FIGURE 6:**
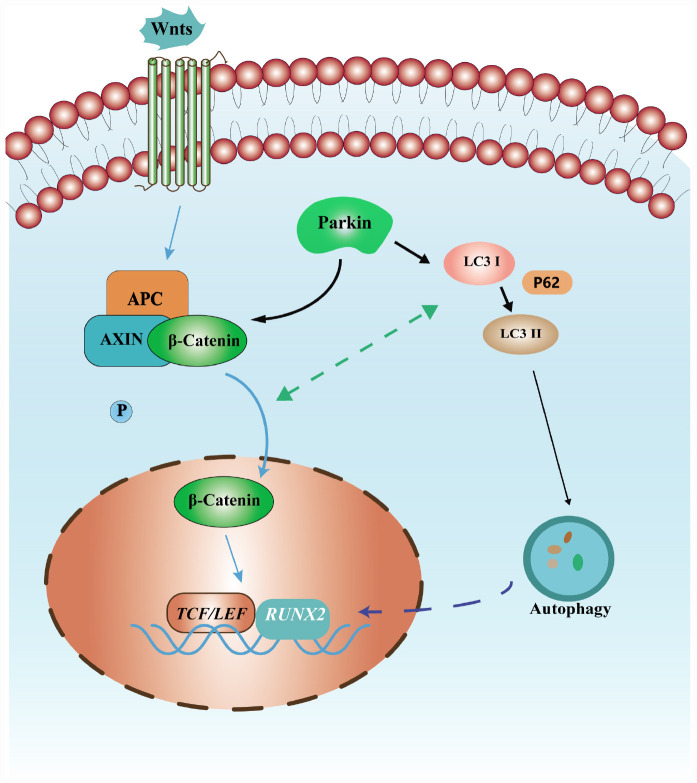
Schematic diagram of the mechanism of osteoblastic differentiation induced by Parkin in this study. Parkin regulates the osteoblastic differentiation of BMSCs via autophagy and β-catenin signaling.

Autophagy is an essential catabolic process that maintains cellular homeostasis by degrading or recycling relevant proteins and organelles (Ma et al., 2018). Conversion of a cytosolic form of LC3 (LC3-I) to the LC3-phosphatidylethanolamine conjugate LC3-II and p62 degradation were greatly enhanced during autophagy (Kabeya et al., 2004). Accumulating evidence indicates that autophagy plays a crucial role in the regulation of bone homeostasis (Nollet et al., 2014; Gomez-Puerto et al., 2016). Autophagy enhances osteoblastic differentiation of BMSCs (Wan et al., 2017; Pan et al., 2019). Autophagy also regulates mesenchymal stem cell properties and senescence during bone aging (Ma et al., 2018). Reduced autophagy decreases osteoblast formation and matrix mineralization and induces significant bone loss (Li et al., 2018). Previous studies demonstrated that Parkin is involved in mitochondrial quality control and autophagy (Narendra et al., 2012; Durcan and Fon, 2015; Seirafi et al., 2015). Parkin regulated mitochondrial quality control in an LC3/ATG-independent manner (Nardin et al., 2016). Parkin was recruited to impaired mitochondria and accelerates their elimination via autophagy. Zhao et al. revealed that NIPA2 increased osteoblast function, which was likely regulated by PTEN induced kinase 1/Parkin-mediated mitophagy in type 2 diabetes and osteoporosis (Zhao et al., 2020). Several studies have indicated that the Wnt/β-catenin signaling pathway is implicated in autophagy (Romero et al., 2018; Chu et al., 2019; Ma et al., 2019). Petherick et al. (2013) revealed that β-catenin suppressed autophagosome formation and inhibited p62 via TCF4. Thus, the relationship between the Wnt/β-catenin signaling pathway and autophagy were addressed in our study. We found that the levels of autophagy may have been activated by the β-catenin agonist. Interestingly, β-catenin expression can be induced by rapamycin. Based on these results, we inferred that β-catenin signaling and autophagy impact one another, resulting in active and complex crosstalk. Parkin regulated osteoblastic differentiation of BMSCs via β-catenin and autophagy signaling pathways (Figure 6).

The present study has several limitations. First, although our results suggest that Parkin mediates the β-catenin and autophagy signaling pathway to regulate osteogenic differentiation of BMSCs, other signaling pathways are also likely involved. Understanding of the detailed crosstalk between the different signaling pathways will require further investigation. Second, our results suggest that Parkin regulates osteoblastic differentiation but a transgenic *Parkin* mouse model will provide more convincing evidence and help further elucidate the underlying mechanisms.

## Conclusion

Based on our data, we found that upregulation of Parkin enhanced the osteoblastic differentiation of BMSCs by upregulation of β-catenin and autophagy signaling.

## Data Availability Statement

The datasets used and/or analyzed during the current study are available from the corresponding author on reasonable request.

## Ethics Statement

The animal study was reviewed and approved by the Animal Ethics Committee of Zhejiang University.

## Author Contributions

WZ, CY, and WL contributed design and funding sources to this study. WZ and MC drafted the manuscript. WH, EC, DX, and ZP did all the *in vitro* and *in vivo* parts of the study. All authors have contributed significantly and read and approved the final manuscript.

## Conflict of Interest

The authors declare that the research was conducted in the absence of any commercial or financial relationships that could be construed as a potential conflict of interest.

## Supplementary Material

MATERIAL S1 Oil Red O staining at days 12 of adipogenic differentiation. Bar = 200 μm. **(A)** Data are expressed as the mean ± standard deviation (SD). NC, BMSCs transfected with negative control adenovirus vectors; OE, BMSCs transfected with Parkin-overexpressed adenovirus vectors; ^∗^*P* < 0.05 vs. BMSCs in the NC group. **(B)** Data are expressed as the mean ± standard deviation (SD) ^∗^*P* < 0.05 vs. group with scramble siRNA.

MATERIAL S2 When *Parkin* was downregulated by siRNA, the expression levels of Parkin, COL1, RUNX2, LC3, p62, β-catenin, and GAPDH protein were determined by WB analysis after osteogenic differentiation for 0, 6, 12, and 24 h. Protein expression levels were normalized to GAPDH. Data are expressed as the mean ± SD of three independent experiments, and one of three independent experiments is shown. ^∗^*P* < 0.05 vs. group with scramble siRNA.

MATERIAL S3 Immunofluorescence for the fracture callus tissues. Expressions of β-catenin and p62 were analyzed by immunofluorescence staining. Bar = 100 μm.

## Abbreviations

BMSCs: Bone marrow-derived mesenchymal stem cells
CCK-8: Cell Counting Kit-8
IF: Immunofluorescence
micro-CT: Microcomputed tomography
RT-qPCR: Quantitative real-time polymerase chain reaction
siRNA: Small interfering RNA
WB: Western blot.
