# Supplementary figures and images for: Upregulation of Parkin Accelerates Osteoblastic Differentiation of Bone Marrow-Derived Mesenchymal Stem Cells and Bone Regeneration by Enhancing Autophagy and β-Catenin Signaling
Source: Front Cell Dev Biol. 2020 Sep 15;8:576104. doi: 10.3389/fcell.2020.576104 (PMC7523089; doi:10.3389/fcell.2020.576104)

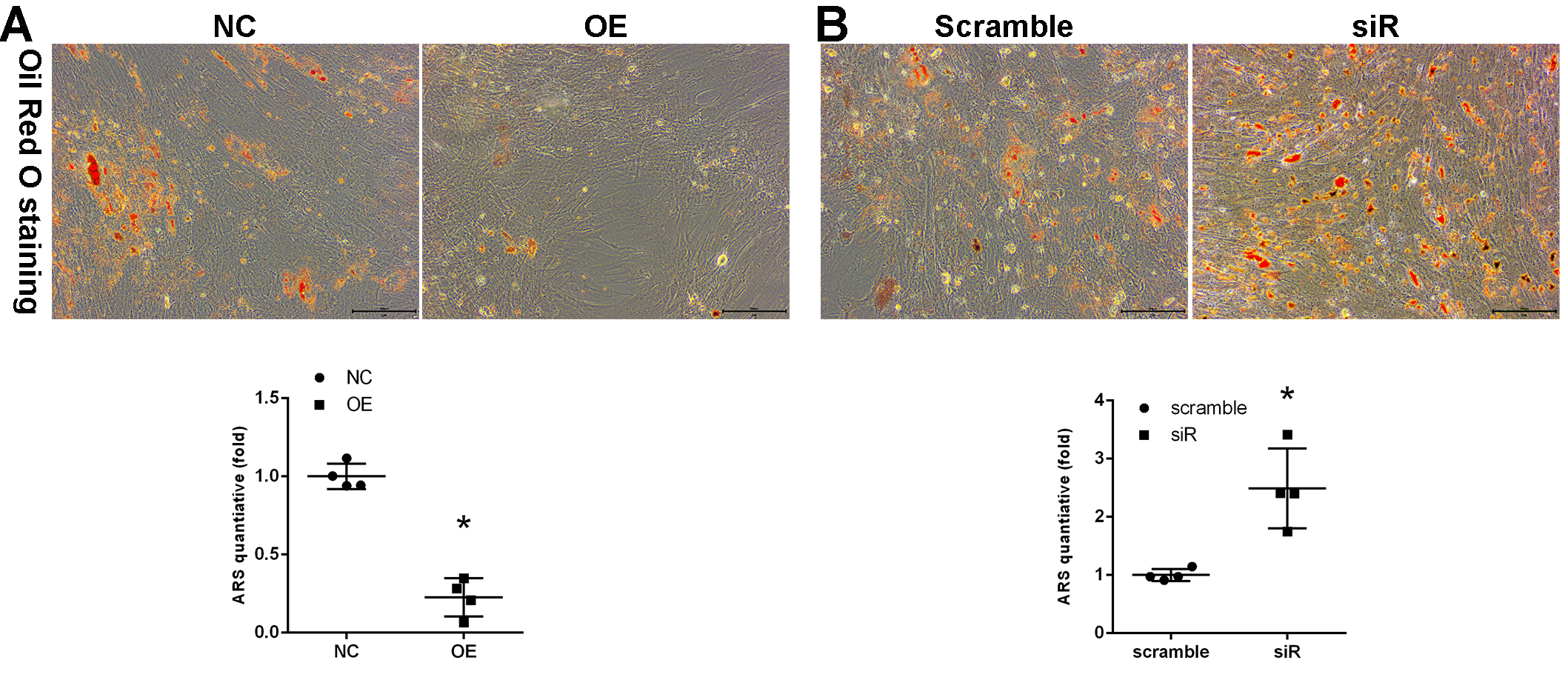

Supplement: MATERIAL S1 — Oil Red O staining at days 12 of adipogenic differentiation. Bar = 200 μm. (A) Data are expressed as the mean ± standard deviation (SD). NC, BMSCs transfected with negative control adenovirus vectors; OE, BMSCs transfected with Parkin-overexpressed adenovirus vectors; ∗P < 0.05 vs. BMSCs in the NC group. (B) Data are expressed as the mean ± standard deviation (SD) ∗P < 0.05 vs. group with scramble siRNA. [file Image_1.JPEG]

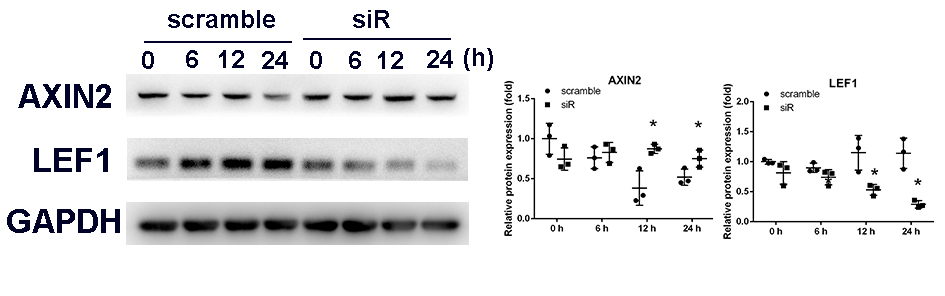

Supplement: MATERIAL S2 — When Parkin was downregulated by siRNA, the expression levels of Parkin, COL1, RUNX2, LC3, p62, β-catenin, and GAPDH protein were determined by WB analysis after osteogenic differentiation for 0, 6, 12, and 24 h. Protein expression levels were normalized to GAPDH. Data are expressed as the mean ± SD of three independent experiments, and one of three independent experiments is shown. ∗P < 0.05 vs. group with scramble siRNA. [file Image_2.JPEG]

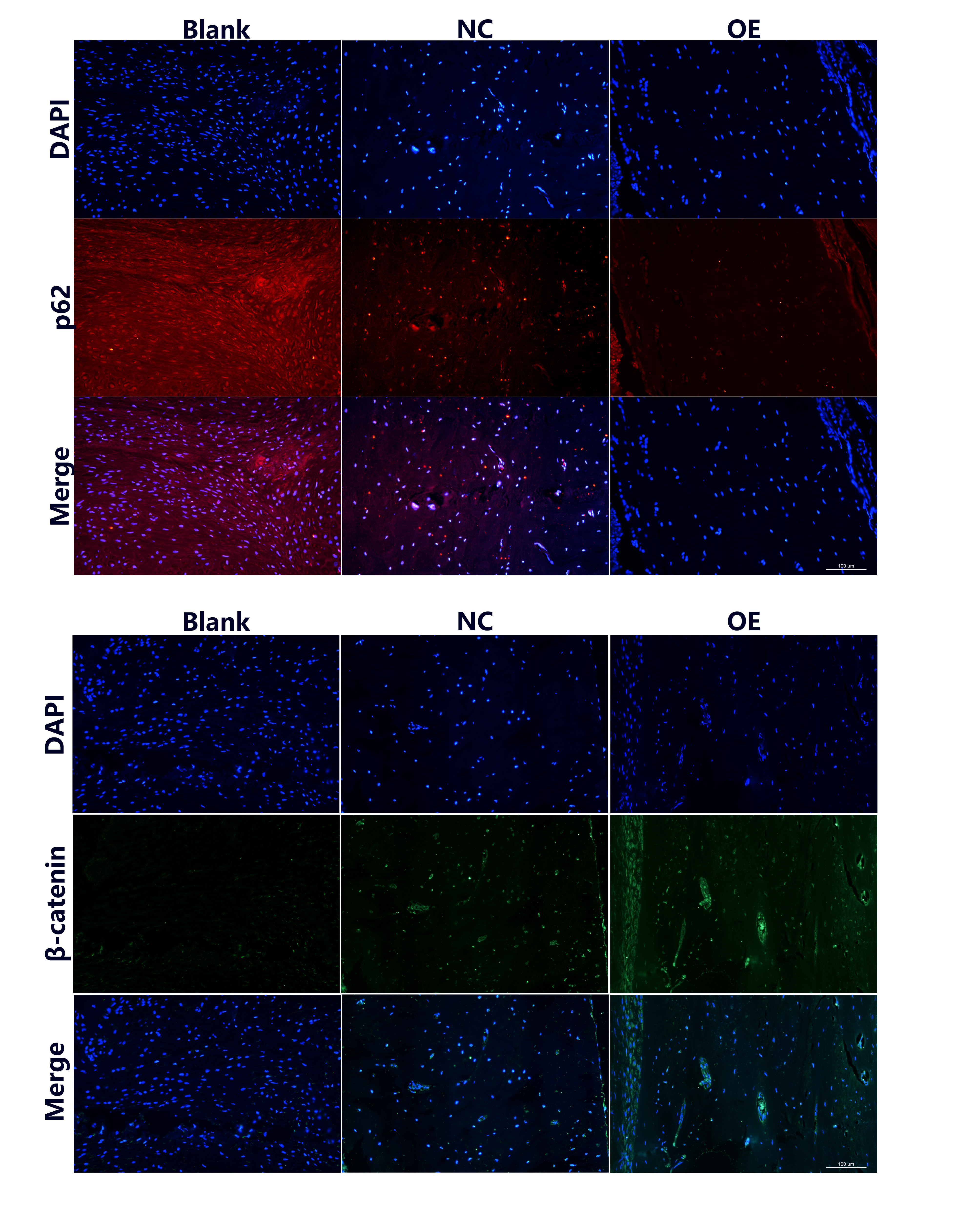

Supplement: MATERIAL S3 — Immunofluorescence for the fracture callus tissues. Expressions of β-catenin and p62 were analyzed by immunofluorescence staining. Bar = 100 μm. [file Image_3.JPEG]
